# Recent advances in PSMA therapy - from beta to alpha

**DOI:** 10.1007/s12149-026-02269-y

**Published:** 2026-08-16

**Authors:** Adrien Holzgreve, Sophie C. Siegmund, Liam Widjaja, Rudolf A. Werner

**Affiliations:** Department of Nuclear Medicine, LMU University Hospital, LMU Medizin, LMU Munich, Munich, Germany

**Keywords:** Prostate-specific membrane antigen (PSMA) radiopharmaceutical therapy (RPT), Radioligand therapy (RLT), Targeted alpha therapy (TAT), Lutetium, Actinium, Astatine, Lead, Theranostics

## The PSMA theranostics concept

The treatment landscape of advanced prostate cancer changed with the adoption of nuclear medicine theranostic applications [1]. The approval of Radium-223 Dichloride for the treatment of symptomatic advanced prostate cancer paved the way, however only targeting the bone reaction to osseous metastases and not addressing primary or recurrent local tumor, lymph node metastases, or visceral metastases [2]. Focusing on prostate-specific membrane antigen (PSMA) as the theranostic target for nuclear medicine applications, however, enabled both visualization and treatment of PSMA-positive disease in prostate cancer, irrespective of the body region or compartment. Early descriptions of the PSMA molecule date back to the 1980’s [3]. The following decades included several approaches to using PSMA as a target for imaging, including antibody-based applications, followed by small molecule PET radioligands for successful PSMA-targeted PET imaging [4]. Few years later, the use of a ¹⁷⁷Lu-labeled PSMA radioligand, however, achieved a remission in a patient with metastatic prostate cancer, i.e. [^177^Lu]Lu-PSMA-617 [5]. This theranostic pair set the stage for a success story in the therapeutic management of patients with advanced disease, shortly following a comparable successful concept for patients with advanced neuroendocrine tumors. Both therapies have been approved around five to ten years ago and revolutionized their fields [6, 7]. Meanwhile, PSMA radiotheranostics have been adopted worldwide, as far as available, and represent a cornerstone in the management of patients with advanced prostate cancer, firmly anchored in clinical guidelines [8].

This article provides an update on established PSMA theranostics using beta-emitting ligands, based on major clinical trial results. It then continues with an overview of more recent developments in the field of PSMA radiotheranostics, focusing on alpha-based approaches, in particular on actinium-based and, subsequently, astatine- and lead-based radiopharmaceutical therapies (RPTs).

## Beta-PSMA RPT: key updates to clinical trials

The clinical development of β-emitting PSMA-targeted RPT, particularly with ^177^Lu-PSMA compounds, has rapidly evolved across different stages of prostate cancer. A series of pivotal trials have had a significant impact on the current therapeutic landscape, leading to an expansion of indications from heavily pretreated metastatic castration-resistant prostate cancer (mCRPC) to earlier disease settings.

The VISION trial was a landmark registration study that established [^177^Lu]Lu-PSMA-617 as a standard of care for patients with advanced mCRPC [9]. In this international, open-label, phase III trial, 1,003 patients underwent ^68^Ga-PSMA PET/CT screening. Of these patients, 831 (82.9%) met all eligibility criteria, including PSMA imaging requirements. Patients were subsequently randomized in a 2:1 ratio to receive RPT (7.4 GBq every 6 weeks for up to 6 cycles) plus protocol-permitted standard of care (SOC) (*n* = 551) or SOC alone (*n* = 280), following prior treatment with at least one androgen receptor pathway inhibitor (ARPI) and one to two taxane regimens. Following the implementation of enhanced educational measures, 581 patients were enrolled in the subsequent phase of the study (385 were administered [^177^Lu]Lu-PSMA-617 and 196 were assigned to the control group). PSMA RPT led to a substantial enhancement in median imaging-based progression-free survival (PFS: 8.7 vs. 3.4 months; HR 0.40; CI 0.29–0.57; *p* < 0.001) and median overall survival (OS: 15.3 vs. 11.3 months; HR 0.62; CI 0.52–0.74; *p* < 0.001). Treatment was generally well tolerated, although grade ≥ 3 adverse events were more frequent in the RPT + SOC arm (52.7% versus 38.0%), primarily due to prolonged treatment exposure. The most prevalent toxicities included hematological adverse events (anemia, thrombocytopenia, lymphopenia) as well as xerostomia and fatigue, with relatively low rates of treatment discontinuation (RPT + SOC arm: 11.9%; SOC arm: not applicable) [9]. This trial led to regulatory approval of [^177^Lu]Lu-PSMA-617 by the FDA and EMA in PSMA-positive mCRPC after prior ARPI and taxane therapy [10].

The randomized phase II TheraP trial implemented a more rigorous biological selection strategy, enrolling 200 patients with mCRPC who had progressed (rising PSA level as defined by Prostate Cancer Working Group 3 (PCWG3) criteria) following docetaxel treatment [11]. A significant distinction from the VISION trial was the implementation of dual-tracer imaging with both PSMA PET/CT and FDG PET/CT for patient selection: patients with discordant lesions (FDG-positive but PSMA-negative) were excluded from the study. Patients were randomly assigned in a 1:1 ratio to receive either [^177^Lu]Lu-PSMA-617 (8.5 GBq every 6 weeks for up to 6 cycles) or cabazitaxel chemotherapy (20 mg / m^2^, every three weeks for up to ten cycles). A total of 291 patients were screened. Following dual tracer PET/CT imaging, 91 patients were excluded from the study. In conclusion, 200 patients were randomly assigned in a 1:1 ratio to receive either cabazitaxel (*n* = 101) or [^177^Lu]Lu-PSMA-617 (*n* = 99). The baseline characteristics exhibited a satisfactory degree of balance, with the vast majority (91%) of subjects having received prior ARPI therapy. A small number of patients did not proceed to treatment, including one patient in the RPT arm who died prior to therapy initiation and several in the cabazitaxel arm who withdrew or became ineligible. The primary endpoint, defined as a PSA response rate of ≥ 50%, was found to be significantly higher in the RPT arm (66% vs. 37%). Furthermore, [^177^Lu]Lu-PSMA-617 significantly delayed disease progression, with progression-free survival HR 0.63 (95% CI 0.46–0.86; *p* = 0.0028). The investigation revealed consistent benefits for radiographic progression-free survival (HR 0.64; *p* = 0.0070) and PSA progression-free survival (HR 0.60; *p* = 0.0017) [11]. The median OS was 16.4 months (95% CI 13.7–19.4) in the [^177^Lu]Lu-PSMA-617 group, as compared to 19.4 months (14.0–21.7) in the cabazitaxel group (HR 0.97; 95% CI 0.70–1.35; *p* = 0.99), with analogous restricted mean survival times (19.1 vs. 19.6 months). It is noteworthy that the survival curves intersected at approximately 24 months, indicating the presence of time-dependent treatment effects and the potential impact of subsequent therapeutic interventions [12]. Toxicity was observed to be favored by [^177^Lu]Lu-PSMA-617, with lower rates of grade ≥ 3 adverse events (33% vs. 53%), particularly reduced neutropenia and febrile neutropenia, while typical RPT-related toxicities included xerostomia, fatigue, and mild hematologic suppression [11].

The open-label, phase III, randomized, controlled PSMAfore trial further expanded the use of PSMA RPT into earlier lines of therapy by enrolling 468 taxane-naïve patients with mCRPC progressing after one prior ARPI following PSMA PET/CT [13]. Patients were randomly allocated (1:1) into two groups: the first group was administered [^177^Lu]Lu-PSMA-617 (7.4 GBq every six weeks for six cycles), while the second group was given a change in ARPI (either abiraterone or enzalutamide). Patients were permitted to crossover upon progression. At the third data cutoff, RPT demonstrated a significant improvement in radiographic PFS, with a hazard ratio of 0.49 (95% CI 0.39–0.61). The median radiographic PFS was 11.60 months (95% CI 9.30–14.19) in the RPT arm, compared to 5.59 months (95% CI 4.21–5.95) in the ARPI change arm. A substantially higher PSA response rate was observed with [^177^Lu]Lu-PSMA-617 (51% versus 17%). At the time of analysis, the median follow-up for overall survival was comparable between groups, at 17.94 months (interquartile range [IQR ] 12.72–22.60) in the RPT arm and 18.45 months (14.59–22.80) in the ARPI change arm. Nevertheless, the OS data are confounded by the high crossover rate, which limits definitive conclusions regarding survival benefit. The safety profile was favorable, with lower rates (36% versus 48%) of grade ≥ 3 adverse events compared to ARPI change, and predominantly mild toxicities including xerostomia, fatigue, and low-grade hematologic effects [13].

The phase III PSMAddition trial is a study that evaluates the role of [^177^Lu]Lu-PSMA-617 in untreated or minimally treated metastatic hormone-sensitive prostate cancer (mHSPC) [14]. This trial is moving earlier in the disease course than other trials. The trial has been designed to determine whether the early integration of RPT can improve long-term outcomes and to ascertain the tolerability of the combination of the ADT/ARPI treatment with intensified systemic therapy. Patients who met the eligibility criteria were required to have at least one PSMA-positive metastatic lesion visible on [^68^Ga]Ga-PSMA-11 PET/CT imaging. In total, 1,144 patients were randomized 1:1 to receive [^177^Lu]Lu-PSMA-617 (7.4 GBq every six weeks for six cycles) plus SOC (ADT + ARPI) (*n* = 572) or SOC alone (*n* = 572). Crossover to the [^177^Lu]Lu-PSMA-617 arm was permitted upon radiographic progression. The study met its primary endpoint, demonstrating a significant improvement in radiographic PFS, with a HR of 0.72 (95% CI 0.58–0.90; *p* = 0.002). At the time of analysis, the median radiographic PFS had not yet been reached in either arm. Preliminary OS data demonstrate a tendency towards enhanced survival in the intervention arm (HR 0.84; 95% CI 0.64–1.13; *p* = 0.125), though statistical significance has yet to be attained, presumably due to the limited follow-up and the crossover design. With regard to safety, grade ≥ 3 events occurred in 51% in the experimental arm versus 43% in the control arm [14].

Finally, the phase II LUNAR trial explores a distinct clinical niche in oligorecurrent, hormone-sensitive prostate cancer (HSPC), addressing the high rate of progression following metastasis-directed therapy alone [15]. This single-center, randomized, open-label study, enrolled patients with 1–5 PSMA PET/CT–positive metastases, who were not on ADT. The patients were stratified by metastatic stage and lesion number, and 92 patients (1:1) were randomized to receive either stereotactic body radiotherapy (SBRT) alone (*n* = 47) or two cycles of neoadjuvant [^177^Lu]Lu-PNT2002 (6.8 GBq per cycle) followed by SBRT (*n* = 45). Following a median follow-up period of 22 months, the incorporation of radioligand therapy resulted in a substantial enhancement in outcomes, with a median PFS of 17.6 months (95% CI 15–not reached) in the intervention arm, as opposed to 7.4 months (95% CI 6.0–13.5) with SBRT alone. This corresponds to a hazard ratio of 0.37 (95% CI 0.22–0.61; *p* < 0.0001). Beyond the primary endpoint, salvage hormonal therapy–free survival was prolonged from 14.1 months to 24.3 months, corresponding to a hazard ratio of 0.40 (95% CI 0.22–0.71; *p* < 0.0001). Reported grade ≥ 3 adverse events were lymphopenia, occurring in 4.8% of patients in the SBRT arm and 6.7% in the combination arm. The results of this study lend support to the hypothesis that the combination of systemic PSMA-targeted RPT with local ablative radiation therapy enhances disease control and delays progression [15].

**Fig. 1:**
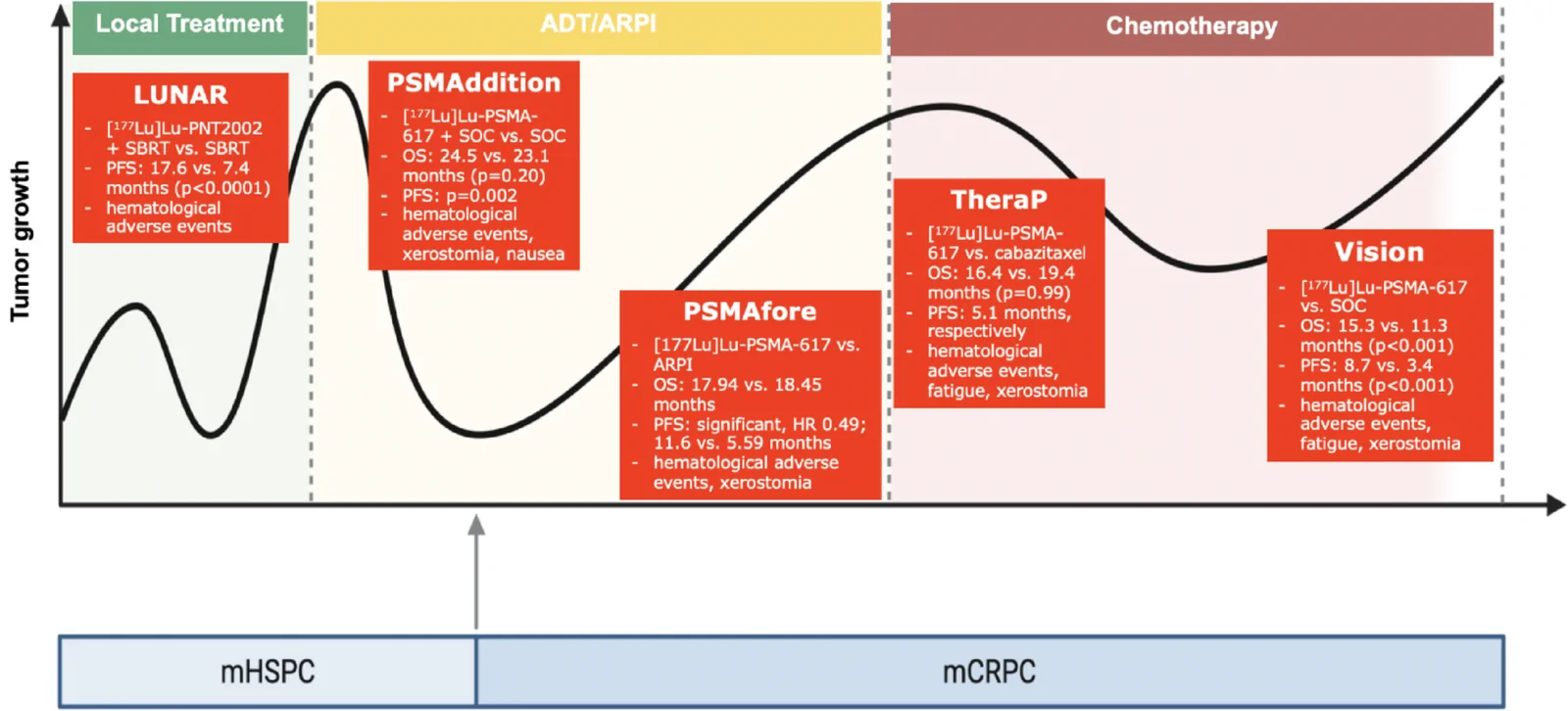
Positioning of key β-emitting PSMA radioligand therapy trials across the prostate cancer disease continuum. This schematic overview provides a comprehensive representation of selected major clinical trials that are evaluating [^177^Lu]Lu-PSMA–based radioligand therapy in relation to disease stage and treatment sequencing. In the context of metastatic hormone-sensitive prostate cancer (mHSPC), the PSMAddition trial explores the efficacy of upfront intensification in combination with androgen deprivation therapy (ADT) and androgen receptor pathway inhibitors (ARPI), while the LUNAR trial assesses the incorporation of radioligand therapy into metastasis-directed stereotactic body radiotherapy in oligorecurrent disease, with the objective of delaying systemic therapy. In the context of metastatic castration-resistant prostate cancer (mCRPC), the PSMAfore trial establishes PSMA RPT as a subsequent treatment following chemotherapy in cases where progression on ARPI therapy has occurred. In contrast, the TheraP trial directly compares radioligand therapy with cabazitaxel, subsequent to docetaxel. The VISION trial establishes PSMA RPT in heavily pretreated patients following ARPI and taxane-based chemotherapy

Collectively, these trials illustrate the rapid expansion of β-emitting PSMA RPT across the prostate cancer disease spectrum (see Fig. 1). From its initial validation in heavily pretreated mCRPC in VISION, through biologically refined patient selection in TheraP, to earlier-line use in PSMAfore and upfront intensification strategies in PSMAddition in mHSPC, as well as combinatorial approaches in oligometastatic disease in LUNAR, PSMA-targeted therapy is increasingly being integrated into personalized and stage-adapted treatment algorithms [9, 11, 13–15].

### ^225^Ac-PSMA RPT: current clinical evidence and perspectives

As summarized above, the β-emitting ^177^Lu-PSMA radioligands have demonstrated significant clinical benefit, including improved OS in randomized trials [9]. However, a subset of patients exhibits primary or late on-set resistance, and most responders eventually experience disease progression. This has led to increasing interest in α-emitting radionuclides, particularly actinium-225 (^225^Ac), as an alternative or complementary therapeutic strategy applying beta-emitters [16]. ^225^Ac is an α-emitting radionuclide with a half-life of approximately 10 days [17]. Its decay chain releases multiple α-particles, leading to high-energy deposition over a very short tissue range (typically < 100 μm) [17]. This high linear energy transfer (LET) results in dense ionization tracks and induces more complex and sustained DNA damage, thereby conferring substantially greater cytotoxicity compared to the β-radiation emitted by ^177^Lu [18]. The production of ^225^Ac is currently limited, representing a significant barrier to widespread clinical use [17]. Sources include decay of thorium-229 and accelerator-based production, both of which face logistical and scalability challenges [17]. Nonetheless with rising need, recent years have witnessed large scale investments into ^225^Ac production, thus by 2032 capacity is expected to be sufficient for at least 2 million patient doses per year [19]. One major advantage of ^225^Ac is, that it can be labeled with commonly used PSMA-ligands, particularly PSMA-617 and PSMA-I&T [16]. Radiochemical stability and dosimetry remain areas of active research, particularly given the complex decay chain of ^225^Ac and the potential redistribution of daughter radionuclides [20].

**Fig. 2:**
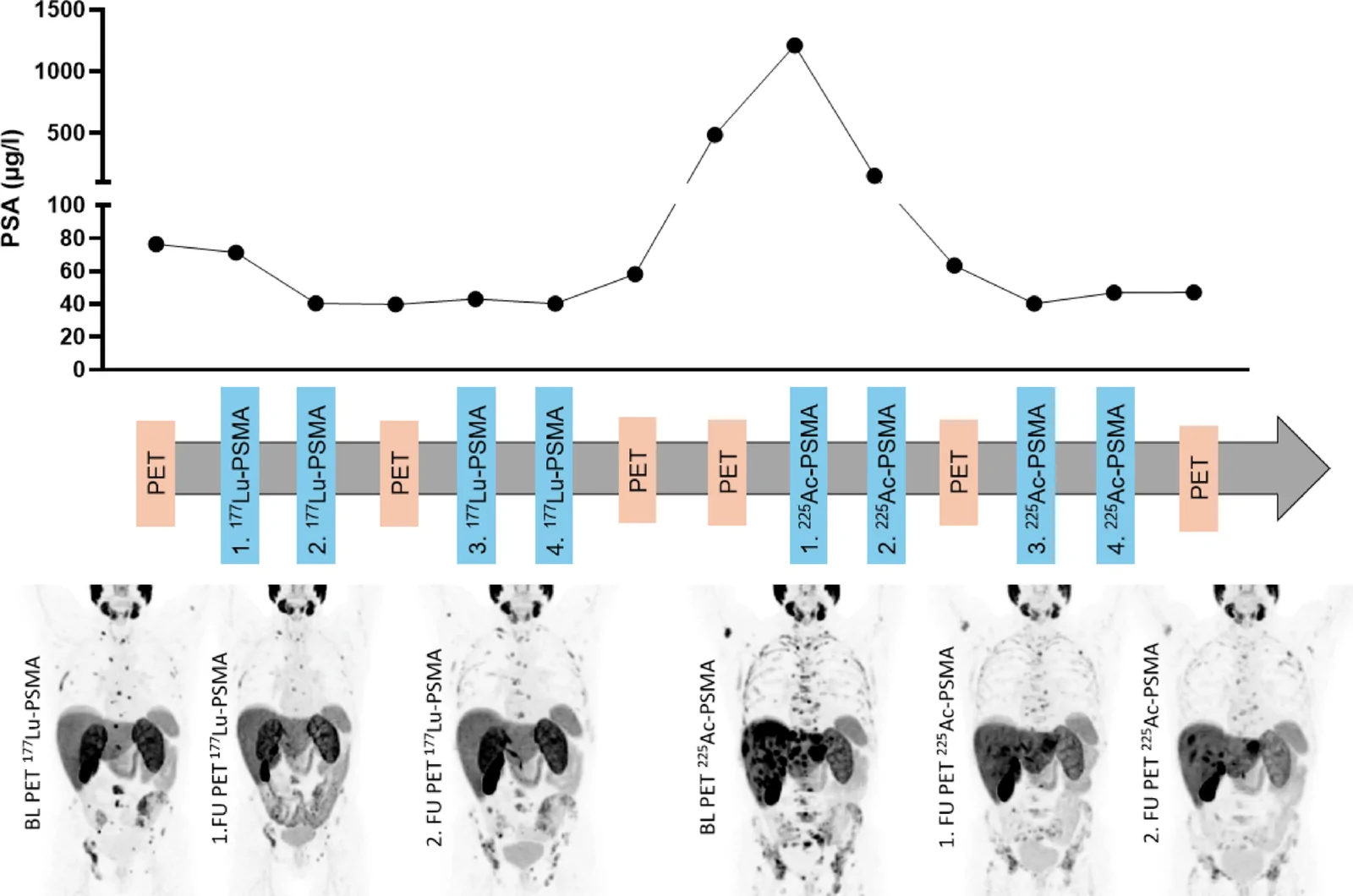
Case illustrating the therapeutic potential of ^225^Ac-PSMA in mCRPC after progression under ^177^Lu-PSMA. The patient initially presented with multifocal osseous metastases on [^18^F]PSMA-1007 PET/CT following androgen deprivation therapy, abiraterone acetate, and docetaxel, with a PSA of 76.5 ng/ml. Treatment with ^177^Lu-PSMA achieved an initial response (PSA nadir 36.1 ng/ml), but subsequent PSA rise and PET/CT after four cycles revealed multifocal progression, leading to discontinuation. Further therapies with carboplatin and enzalutamide were ineffective, and PSA increased to 1,211 ng/ml with newly developed disseminated liver metastases. As a last-line approach, ²²⁵Ac-PSMA was initiated and demonstrated marked efficacy. PSA declined to 40.3 ng/ml with imaging response after two cycles and remained stable (47.1 ng/ml) after four cycles, with only minor progression on PET. PET = positron emission tomography; PSA = prostate-specific antigen; Lu = lutetium; Ac = actinium; PSMA = prostate-specific membrane antigen; BL = baseline; FU = follow-up

Kratochwil et al. were among the first to demonstrate the high therapeutic potential of targeted alpha therapies (TAT) illustrating a case report of a patient with widespread disease progression under ^177^Lu-PSMA and subsequent complete response after 3 cycles of ^225^Ac-PSMA RPT [21]. Further building up on these results, early clinical studies of ^225^Ac-PSMA RPT were conducted in heavily pretreated mCRPC patients who had exhausted standard treatment options [22–24]. The largest retrospective study so far, the WARMTH Act conducted by Sathekge et al., included 488 men with heavily pretreated mCRPC at seven centers [25]. In this study 57% of the included patients achieved a PSA-response (PSA-decline ≥ 50%) compared to the 46% in the VISION trial under ^177^Lu-PSMA [9, 25]. Of note, median progression-free survival (PFS, 7.9 months) and overall survival (OS, 15.5 months) were also highly promising [25]. In a large meta-analysis pooling the results of eighteen studies with 1,155 patients, ^225^Ac-PSMA RPT PSA-response rates ranged from 26 to 91% [16]. Interestingly, data suggest that efficacy was particularly impressive when ^225^Ac-PSMA RPT was conducted earlier in the treatment course e.g., with a PSA-response rate of 82% as a first line therapy for mCRPC [16]. Nonetheless, Feuerecker et al. reported that even in patients with previous progression under ^177^Lu-PSMA, PSA-response can be achieved in 65% [24]. A recent study also systematically analyzed pretherapeutic PSMA-PET/CTs and demonstrated that whole-tumor mean Standardized Uptake Value (SUV_mean_) is predictive for PFS, whilst the PSMA-Total Lesion Quotient (PSMA-Tumor Volume divided by SUV_mean_) predicts OS [26]. Thus, PSMA-PET/CT may be used for individualized patient selection [26]. A case illustrating the therapeutic potential of ^225^Ac-PSMA after progression under ^177^Lu PSMA is shown in Fig. 2.

Despite initial concerns that the increased therapeutic potency of α-emitting radioligands might be associated with higher toxicity, ^225^Ac-PSMA RPT has mainly demonstrated a favorable safety profile in patients with mCRPC [16]. Due to physiological PSMA expression in the salivary glands, xerostomia is one of the most common adverse effects of PSMA-targeted RPT, particularly with ^225^Ac-based therapies [25, 27]. In the WARMTH Act, the incidence of xerostomia was dose-dependent, with up to 91% of patients reporting symptoms after the third treatment cycle [25]. Importantly, however, dose-limiting xerostomia remained rare, occurring in only 2% of patients [25]. Additional toxicities include potential bone marrow suppression and renal impairment [25]. In the WARMTH Act cohort, mild to moderate anemia was observed in 67% of patients and renal functional impairment in 51% [25]. Notably, reflecting the advanced and heavily pretreated disease stage typical for this patient population, the majority of these abnormalities were already present prior to initiation of ^225^Ac-PSMA RPT (e.g., preexisting anemia in 62%). Severe treatment-related adverse events were uncommon, with new-onset grade ≥ 3 anemia occurring in only 8% of patients and grade ≥ 3 renal impairment in approximately 1% [25]. Overall, these findings support the notion that ^225^Ac-PSMA RPT may be a clinically manageable and well-tolerated treatment option, even in a late-line setting.

The field of ^225^Ac-PSMA RPT is rapidly evolving, with current research focusing on optimizing efficacy, safety, and its integration into treatment algorithms [28]. A particularly promising approach is a tandem RPT combining ^177^Lu- and ^225^Ac-PSMA [29]. While α-particles from ^225^Ac provide highly potent cytotoxic effects due to their high LET, their short tissue range may limit efficacy in bulky disease [29]. In contrast, β-emitting ^177^Lu offers a greater tissue penetration and crossfire effect. Combining both radionuclides may therefore exploit complementary properties and enhance therapeutic efficacy [29]. Early clinical data support this concept, demonstrating disease control rates of approximately 60% in heavily pretreated patients [29]. Importantly, tandem approaches may also reduce xerostomia by lowering the required α-activity [30]. In parallel, several prospective clinical trials are currently underway to further define the role of ^225^Ac-PSMA RPT within existing treatment algorithms [28]. Among these, the PSMAcTION trial is of particular relevance [31]. This ongoing phase II/III randomized controlled trial, initiated in February 2025 and expected to be completed in 2028, directly compares ^225^Ac-PSMA RPT with standard-of-care therapies in patients with mCRPC who have progressed under prior ^177^Lu-PSMA [31]. As one of the first large-scale randomized studies in this field, PSMAcTION is anticipated to provide pivotal evidence regarding both efficacy and safety in patients having experienced progression under ß-emitting PSMA RPT, thereby addressing a major gap in the current literature [31]. Another highly innovative study is the ANDROMEDA trial, which represents the first prospective head-to-head comparison of ^225^Ac-PSMA and ^177^Lu-PSMA in an earlier disease setting [32]. In this trial, both radioligand therapies are administered in combination with metastasis-directed stereotactic body radiotherapy in patients with oligometastatic prostate cancer [32]. This design reflects a broader shift in the field toward evaluating PSMA-targeted RPT in less advanced disease stages and in multimodal treatment concepts [32]. The results of the ANDROMEDA trial are expected to provide important insights into the comparative efficacy of α- versus β-emitting radioligands and may help to define their optimal sequencing or combination [32]. Taken together, these ongoing studies highlight a transition from retrospective analyses toward prospective, randomized evidence generation. They are likely to play a key role in further establishing ^225^Ac-PSMA RPT, not only as a salvage option after β-emitting therapies but potentially as an integral component of earlier and more individualized treatment strategies in prostate cancer.

### ^211^At-PSMA RPT: current state of a promising novel theranostic approach

While ^225^Ac has pioneered alpha-based PSMA-RPT and, meanwhile, is investigated in large clinical trials, new radionuclides for alpha-based PSMA-RPT are also under investigation. Among others, lead-212 (^212^Pb) and astatine-211 (^211^At) emerged as promising new alpha-emitting radionuclides in the context of PSMA-RPT.

The physical properties of ^211^At bring several advantages. With 7.2 h, ^211^At has a considerably shorter half-life compared to ^225^Ac with 238.1 h [33]. This short half-life and the simple decay chain of ^211^At provide a clearer picture of its distribution and further re-distribution of daughter nuclides within the body, and they facilitate waste management, as long-term radioactivity is not a concern [33]. ^211^At can be produced with a 30-MeV cyclotron using a natural bismuth-209 (^209^Bi) target, abundant on earth [33]. Thus, advanced technical prerequisites are placed on the production facilities, but this mechanism also renders ^211^At production less dependent of scarce raw materials, as sometimes encountered with other alpha-based RPTs. Therefore, in principle, ^211^At is easily scalable via cyclotron-based manufacturing, overcoming the concern of unreliable supply chains for alpha-based RPT.

The radiosynthesis of ^211^At-eligible PSMA ligands is now established and has been described in several papers [34, 35]. One of the pivotal candidates for ^211^At-based PSMA RPT is PSMA-5. The molecular structure of [^211^At]PSMA-5 has been directly derived from the established diagnostic PET compound [^18^F]PSMA-1007, sharing favorable pharmacokinetic properties for theranostic application [36]. Other ^211^At-based PSMA radioligands with streamlined and scalable radiolabeling method include [^211^At]YF2 [35].

Preclinical evaluation of [^211^At]PSMA-5 suggests its efficacy and safety. The half-maximal inhibitory concentration of [^211^At]PSMA-5 in LNCaP prostate adenocarcinoma cells was 0.32 nM with PSMA-617 as the blocking agent [37]. In vivo in mice, [^211^At]PSMA-5 showed a blood cell association rate of 29.3% and a plasma protein binding rate of 62.0% [33]. In vivo in cynomolgus monkeys, [^211^At]PSMA-5 showed blood cell association rate of 18.2% and a plasma protein binding rate of 77.0% [33]. In both species, the compound remained stable in the blood without relevant radiolabeled metabolites detectable, neither in plasma nor urine. Compared to other tested compounds such as [^211^At]PSMA-1 and [^211^At]PSMA-6, [^211^At]PSMA-5 exhibited the highest tumor retention with 40.7 ± 2.6%ID/g at 24 h, resulting in a measurable treatment effect on tumor growth [38]. Some transient toxicities such as mild leukopenia 24 h post-administration in monkeys were noted, yet no significant myelosuppression or renal dysfunction in mice nor any durable toxicities were recorded. Also, histological analysis revealed no major abnormalities. Regenerated tubules were detected in the kidneys at 3 and 6 weeks after the administration of [^211^At]PSMA-5 [38]. In sum, no irreversible or severe toxicities associated with [^211^At]PSMA-5 (up to 35 MBq/kg) were noted in preclinical evaluation.

Initial clinical experiences with ^211^At are not confined to prostate cancer but include patients with brain tumors, ovarian cancer, and thyroid cancer [39–41]. In a pivotal phase 1 trial (“Alpha-T1 Trial”) in eleven patients with advanced differentiated thyroid cancer, Watabe et al. performed the first-in-human dose escalation study of [^211^At]NaAt following a modified 3 + 3 design to evaluate its safety and preliminary efficacy [41]. Meanwhile, preliminary first-in-human data is available also for ^211^At-based PSMA RPT in patients with prostate cancer. Watabe et al. published a first-in-human SPECT/CT image of [^211^At]PSMA-5 in comparison to pre-therapeutic [^18^F]PSMA-1007 PET imaging, illustrating a concordantly high accumulation of the radioligands in tumor lesions and a concordant physiological accumulation in the salivary glands, liver, spleen, small intestine, and kidneys, providing a proof-of-concept for this alpha-based theranostic approach. This research group also currently runs the first-in-human phase 1 investigator-initiated clinical trial on [^211^At]PSMA-5 in patients with prostate cancer at the University of Osaka Hospital (NCT06441994) [33]. Recruitment started in May 2024 with an overall 15 patients planned to be enrolled by March 2027. Eligible are patients with progressive mCRPC who have previously received androgen receptor pathway inhibitors (enzalutamide, apalutamide) or a CYP17 inhibitor (abiraterone) and taxane-based chemotherapy, if clinically feasible. The trial design includes sequential dose escalation, aiming to determine an optimal dose for a subsequent phase 2 trial. The primary study endpoints include the incidence of dose-limiting toxicities and treatment-related adverse events, the secondary study endpoints include pharmacokinetic measurements, biodistribution, dosimetry, and preliminary efficacy as assessed by PSA response rates and imaging-based evaluation using CT and PET [33]. Other pivotal first-in-human data on ^211^At-based PSMA RPT were recently published by Wu et al. using [^211^At]YF2 in a phase 0 microdose study (29.6–44.4 MBq) [42]. In three patients with mCRPC and PSMA-positive metastases confirmed by previous PSMA PET imaging, [^211^At]YF2 was well tolerated; no adverse events of grade 3 or higher were recorded for 7 days post injection. While early after injection a high uptake was noted in kidneys, bladder, salivary glands, and the thyroid, this was then followed by a rapid clearance. Pronounced uptake was retained in metastases even at 24 h post injection (with an estimated average dose up to 31.4 mGy/MBq per lesion), supporting further clinical investigation of this compound as well [42].

Overall, ^211^At-based approaches are a promising novel development in the field of alpha-based therapies, with the potential to substantially diversify and advance the field of PSMA RPT in prostate cancer.

### ^212^Pb-PSMA RPT

Another novel approach to targeted alpha-based PSMA-RPT is the use of lead-212 (^212^Pb) as the therapeutic nuclide. While it shares some advantages with astatine over actinium, including a significantly shorter half-life of 10.6 h compared to 238.1 h for ^225^Ac, ^212^Pb differs from ^211^At in some respects [43]. ^212^Pb is a heavy metal and not a halogen, potentially favoring in vivo stability and contributing to a different profile of potential organs-at-risk. As opposed to ^211^At, which is produced at cyclotrons, ^212^Pb can be extracted continuously from local thorium-228 generators. Due to its high gamma decay, ^212^Pb facilitates post-therapeutic imaging and dosimetry in the context of alpha-based PSMA-RPT [43].

Two early trials on ^212^Pb-based PSMA RPT include the ARTISAN (NCT07214961) and the TheraPb (NCT05720130) trials. ARTISAN is a phase 1 trial investigating AB001 ([^212^Pb]Pb-NG001) in patients with mCRPC in ^117^Lu-naïve and post-^117^Lu cohorts, TheraPb is a phase 1/2 trial in patients with mCRPC while the phase 2 also includes the mHSPC setting, chemotherapy-naïve and post-^117^Lu [44, 45]. A first-in-human case publication of post-therapeutic SPECT/CT images out of the TheraPb cohort demonstrated concordant tumor biodistribution with low salivary gland uptake and rapid kidney clearance compared to pre-therapeutic PSMA-PET/CT [46]. Data of the completed dose escalation phase of the TheraPb trial were presented at the ESMO Congress 2025 [47]. 16 patients have been included in the dose escalation phase. No grade 3 or higher treatment-related adverse events were noted. The most common treatment-related adverse events were xerostomia (75% overall, 69% grade 1 for more than 7 days), nausea (50%), and fatigue (44%). No myelosuppression or renal impairment was noted. In 75% of patients receiving ≥ 120 MBq, a PSA50 response with corresponding decline of PSMA-avid tumor volume on PET was noted [47]. Overall, these preliminary data support the approach due to the favorable safety profile and promising anti-tumor activity.

## Conclusion

In summary, PSMA-based radioligand therapies are an established treatment option for patients with advanced prostate cancer. Evidence on patient benefit exists particularly for beta-emitting ligands, which are already approved for mCRPC and are increasingly being investigated even in earlier stages of the disease, including mHSPC. However, since this treatment is not a cure and has certain limitations, new approaches such as alpha-emitting radioligand therapies are emerging. Current investigations will determine the efficacy and toxicity of those radionuclides, e.g., in patients that have progressed under ß-emitters.
